# Investigating the role of maternal heart rate variability in the onset of labor

**DOI:** 10.3389/fmed.2025.1659620

**Published:** 2025-09-17

**Authors:** Namareq Widatalla, Emerson Keenan, Marimuthu Palaniswami, Ahsan Khandoker

**Affiliations:** ^1^Khalifa University, Abu Dhabi, United Arab Emirates; ^2^Department of Electrical and Electronic Engineering, The University of Melbourne, Parkville, VIC, Australia; ^3^Department of Obstetrics Gynaecology and Newborn Health, The University of Melbourne, Heidelberg, VIC, Australia; ^4^Department of Biomedical Engineering Biotechnology, Healthcare Engineering Innovation Group (HEIG), Khalifa University, Abu Dhabi, United Arab Emirates

**Keywords:** heart rate variability, labor onset, electrocardiogram, parasympathetic & sympathetic activity, electrohysterography

## Abstract

There are currently no measures to accurately predict the onset of labor at term. Currently, the onset of labor is anticipated based on the estimated due date (EDD), which is derived from the day of the last menstrual period or ultrasound-based anatomical information. However, the EDD is not intended to identify physiological factors which may result in the early onset of labor. Therefore, there is a need to identify potential biomarkers that are associated with the onset of labor to accurately predict the timing of delivery. In this exploratory study, we investigated the associations between maternal RR interval (mRRI), maternal heart rate variability (mHRV) features, and the onset of labor. A total of 37 participants were analyzed, including 25 with Electrohysterogram (EHG)-derived signals (age: 28 ± 5.9 years; gestational age (GA): 34 ± 2.7 weeks) and 12 with non-invasive electrocardiogram (NIFECG)-derived signals (age: 32 ± 4.5 years; GA: 38 ± 1.5 weeks). The association of mHRV with the onset of labor was quantified by calculating correlations with time to delivery, defined as the difference between GA at recording and GA at delivery. Correlation analysis revealed that several standard mHRV indices showed strong associations (*r* > 0.5) with time to delivery.

## Introduction

A birth's due date is usually surrounded by uncertainty. Ideally, delivery is expected to occur at 40 weeks of gestation; however, only about 5% of pregnant women give birth precisely at 40 weeks (1, 2). A pregnancy lasting 37–42 weeks is considered full-term, whereas delivery before 37 weeks or after 42 weeks is typically associated with fetal complications and preeclampsia (2). Because the onset of labor is difficult to predict in the absence of well-defined clinical symptoms, clinicians may opt for induction or cesarean section if waiting for spontaneous labor could pose risks. Nevertheless, such decisions are challenging, and inappropriate timing of induction or cesarean delivery may adversely affect fetal development (3, 4).

Currently, the onset of labor is primarily estimated using the estimated due date (EDD), which is calculated with Naegele's rule, 40 weeks after the first day of the last menstrual period. However, EDD based on Naegele's rule is prone to errors due to menstrual cycle irregularities and inaccurate recall of the last menstrual period (2). Anatomical ultrasound is also used to estimate the onset of labor by measuring fetal biparietal diameter, abdominal circumference, and head circumference (3). While ultrasound-based estimation is generally more accurate than Naegele's rule (3, 4), its reliability depends on the timing of the scan.

Identifying potential markers for the onset of labor could enhance pregnancy management and improve delivery outcomes (4, 5). The transition from pregnancy to labor is thought to be measurable through autonomic activity, which reflects neuroendocrine changes (2). Erickson et al. (2) collected physiological data from pregnant women using a multimodal smart ring worn from 30 weeks of gestation until delivery. The recorded data, including sleep, respiration, heart rate (HR), and heart rate variability (HRV), were incorporated into a model to estimate the likelihood of labor onset occurring before or after the clinically estimated due date. Erickson et al. (2) reported significant correlations between these physiological metrics and time to delivery. Changes in HRV during pregnancy have also been documented in previous studies and are thought to reflect alterations in autonomic nervous system (ANS) activity (6–9). Based on the previously mentioned associations between pregnancy and HRV, we aimed here to investigate the association between HRV and time to delivery. We believe that exploring such associations will facilitate pregnancy management by means of HRV measurement.

To our knowledge, few studies (2, 10) have directly examined the correlation between maternal HRV and the timing of labor onset. Prior work has used wearable-derived physiological metrics that typically provide only time-domain estimates of HRV, limiting their ability to capture frequency-domain indices such as high frequency (HF) and low frequency (LF) to HF ratio (LF/HF) that reflect distinct autonomic processes. In contrast, our study uses beat-to-beat R–R intervals derived from maternal electrocardiogram (ECG) and electrohysterography (EHG) recordings, enabling a detailed analysis of both time- and frequency-domain HRV indices.

Fetal HRV has been extensively investigated as a marker of fetal wellbeing, development, and intrapartum complications (7, 11, 12). However, its role in predicting the timing of labor onset has not been established. A major reason is the challenge of reliably acquiring longitudinal fetal HRV data. Continuous or repeated fetal ECG recordings are technically demanding, often affected by low signal quality, and not routinely feasible across gestation (11, 13, 14). In contrast, maternal HRV can be measured more consistently and non-invasively, making it a more practical potential biomarker for exploring associations with labor onset.

## Methods

### Data description

Two datasets were used in this study. The first dataset was obtained online from the Icelandic 16-electrode Electrohysterogram (EHG) Database (Nordic database). The data from this dataset was collected at Akureyri Primary Health Care Centre and Landspitali University Hospital after obtaining Informed consent from every participant. The study protocol was approved by the National Bioethics Committee in Iceland (VSN 02-006-V4). More details about this study are mentioned in (15). Briefly, a total of 122 16-electrode EHG recordings were collected from 45 pregnant women by using a sixteen-channel multi-purpose physiological signal recorder (Embla A10) with a 200 Hz sampling rate. In the Nordic database, multiple recordings were collected from 32 participants, resulting in repeated records for some subjects. Repetitive recordings were excluded, and only those with visible R peaks or minimal visual noise were considered for analysis. Ultimately, one recording per subject was selected, resulting in a total of 45 recordings out of 122. The duration of the recordings ranged from at least 8 min to a maximum of 85 min.

**Figure 1:**
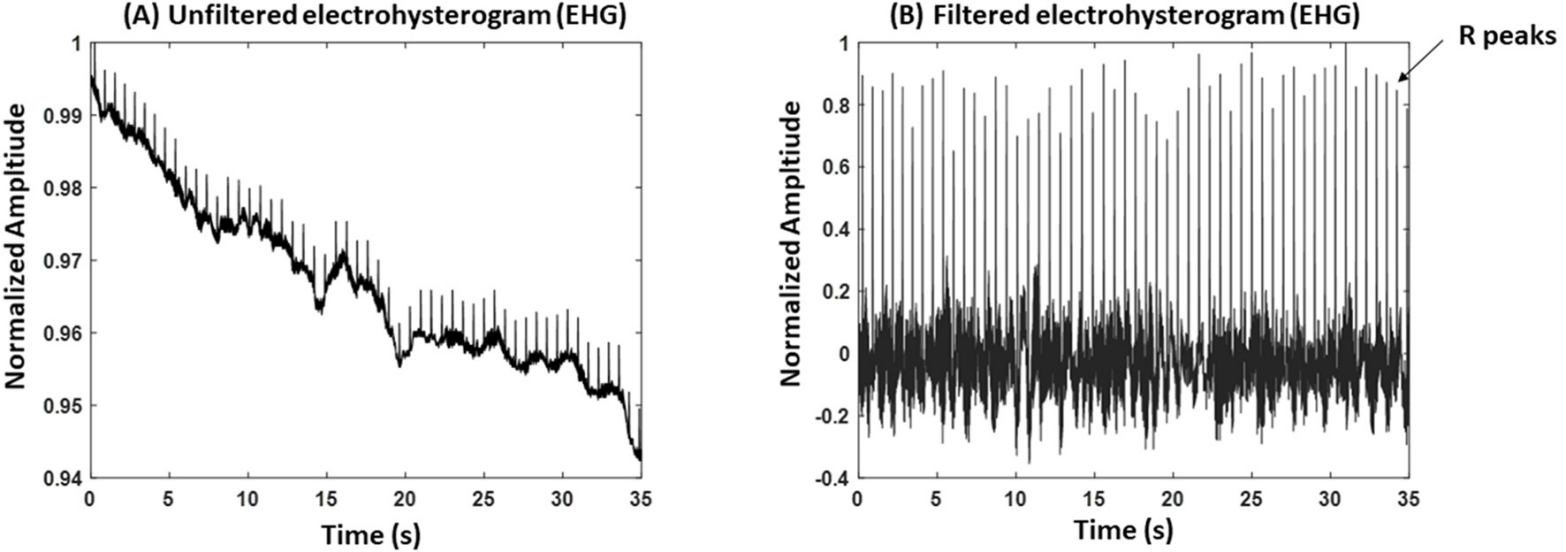
An example of an electrohysterogram (EHG). **(A)** An unfiltered EHG signal. **(B)** A filtered EHG signal. The record belongs to a participant with gestational age (GA) = 36 weeks and 5 days, age: 30 years old.

From the Nordic database, we excluded participants who delivered for non-physiological reasons (induction: *n* = 4; cesarean section: *n* = 6) and those with poorly detectable R peaks for a continuous 5 min segment in the EHG signals (*n* = 10). After exclusion, the total number of participants analyzed from the Nordic database was 25. Maternal electrocardiogram (ECG) noise in EHG recordings is common (16); therefore, we inspected the EHG records for the presence of R peaks to calculate HRV, as illustrated in Figure 1. The final dataset included 25 EHG recordings (maternal age: 27 ± 5.7 years; gestational age (GA): 35 ± 2.9 weeks).

**Figure 2:**
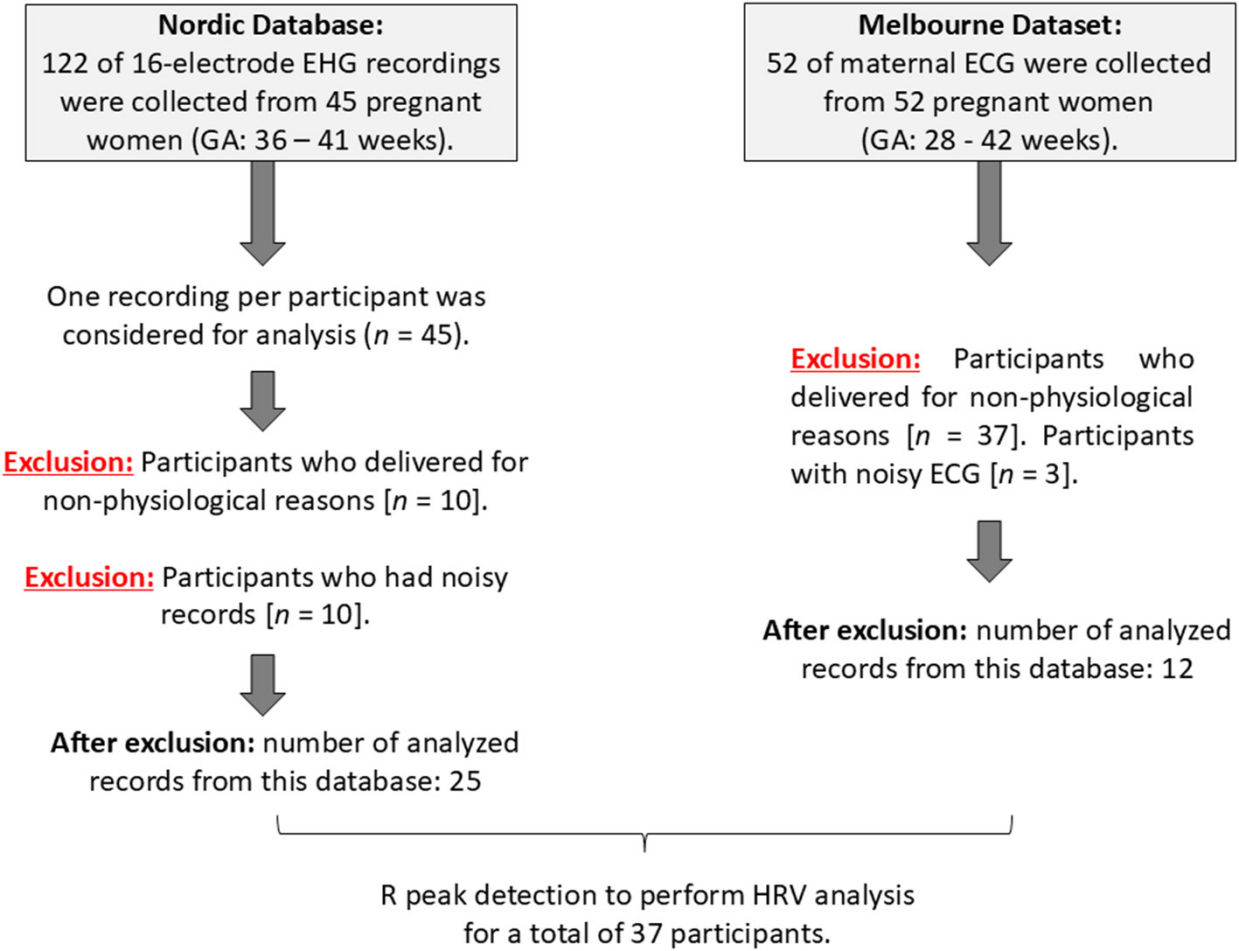
Summary of data analysis of the Icelandic 16-electrode Electrohysterogram (EHG) Database (Nordic database) and the Melbourne dataset.

The second dataset was obtained from a study that was conducted at the Mercy Hospital for Women in Heidelberg, VIC, Australia. Fifty-two women between 18 and 45 years of age with pregnancies between 36 to 41 weeks GA were recruited. The study was approved by the Mercy Health Human Research Ethics Committee (Approval No. 2018-046), and written informed consent was obtained from each participant. The purpose of the study was to record non-invasive fetal and maternal ECG for another project. Here, we are using maternal (mECG) only. For each participant, 30 min of ECG data were recorded at a 1,000 Hz sampling rate and a 24-bit depth using the ADS1299 biopotential amplifier (Texas Instruments, OpenBCI). We excluded pregnancies that were delivered for non-physiological reasons. There were 40 pregnancies delivered for non-physiological reasons, 13 with elective/planned cesarean labor, and 24 with induction. Due to the latter exclusions, 12 records of mECG were considered for analysis, (age: (32 ± 4.2) years old, GA: (38 ± 1.7) weeks). Figure 2 presents a summary of data analysis for each dataset.

### R peak detection and HRV analysis

Despite differences in recording modality (EHG-derived maternal ECG at 200 Hz vs. direct maternal ECG at 1,000 Hz), identical preprocessing steps were applied across both datasets, including filtering, R-peak detection, artifact correction, and HRV extraction from 5 min segments. All RR interval (RRI) values were expressed in milliseconds, ensuring that HRV indices were standardized and directly comparable between datasets.

Figure 1A shows an example of a raw EHG recording, where maternal R peaks are visible due to ECG interference in the abdominal signal. To extract R peaks, we first applied a high-pass filter to reduce baseline drift and low-frequency uterine activity. The filtered signal was then amplitude-normalized (scaled to a maximum of 1) to standardize peak detection (Figure 1B). R peaks were identified using MATLAB's “findpeaks” function, with thresholds set relative to the normalized amplitude to minimize false detections. Detected peaks were visually inspected, and segments with excessive noise or unclear peaks were excluded from analysis. The same methods were applied to detect R peaks from ECG signals.

All analysis described in this study was conducted in MATLAB. To calculate HRV, we selected 5 min from ECG and EHG records. The beginning of the record was the favored choice to select the 5 min; however, the next 5 min segments were selected in case detection of R peaks proved to be difficult in the first 5 min segment due to noise. A 5 min length was used because it is the minimum recommended length for frequency-based HRV analysis (17).

RRI series were corrected manually for ectopic beats, and then time-based and frequency-based HRV were calculated. Manual correction of the RRI series was performed by calculating the mean of consecutive ectopic beats and then dividing by the total number of the same beats. For time-based HRV, the standard deviation of normal RRI (SDNN) and the root mean square of successive differences between normal beats (RMSSD) were calculated. Frequency-based HRV was calculated by using the by the following bands (17): very low frequency (VLF): [0.0033–0.04] Hz, LF: [0.04–0.15] Hz, high frequency (HF): [0.15–0.4] Hz.

### Correlation analysis

To estimate the association of the onset of labor with maternal RRI and HRV, time to delivery was calculated. The time to delivery was calculated by subtracting GA at recording time from GA at delivery. Because the variables did not follow normal distribution based on the Lilliefors test (MATLAB), we used Spearman correlation analysis.

## Results

Table 1 presents a summary of participant demographics for each database or dataset. Melbourne participants had higher age, BMI, and GA (recording) compared to the Nordic database. In Table 2, time-domain measures showed that RMSSD (25 ± 13) ms was significantly negatively correlated with time to delivery [*r* = −0.50, *p* = 0.0016, (CI: −0.75 to – 0.18)], while SDNN (44 ± 14) ms and RR interval (677 ± 79) ms did not show significant associations (*p* > 0.05). For the frequency-based HRV metrics, HF power (5.2 ± 1.0) has a significant negative correlation with time to delivery [*r* = −0.42, *p* < 0.009, (CI: −0.70 to – 0.10)], indicating reduced parasympathetic activity as delivery approached. In contrast, LF/HF ratio (0.69 ± 0.71) was positively correlated with time to delivery [*r* = 0.55, *p* = 0.0004, (CI: 0.23–0.78)], suggesting increased sympathetic dominance. Other frequency components, such as LF (5.9 ± 0.60) and VLF (6.7 ± 0.64), were not significantly associated with labor onset (*p* > 0.05).

**Table 1:** Demographics summary.

**Feature**	**Nordic database**	**Melbourne dataset**
Count	25	12
	**Mean** ±**SD**	**Mean** ±**SD**
Age (Years old)	27 ± 5.7	32 ± 4.1
BMI (Kg/m^2^)	29 ± 4.1	31 ± 11
GA recording (weeks)	35 ± 2.9	38 ± 1.7
GA delivery (weeks)	40 ± 1.2	39 ± 1.3
Analyzed signal	Electrohysterogram (EHG)	Electrocardiogram (ECG)

BMI, body mass index; GA, gestational age.

**Table 2:** Correlations between HRV metrics and gestational measures.

**Feature**	**Mean ±SD**	**GA at the time of recording (29–41) weeks**	**GA at the time of delivery (37–41) weeks**	**Difference between GA delivery and recording (0–9.3) weeks**
**Linear time-based HRV metrics**				
RR (ms)	677 ± 79	*r* = 0.17 [CI: – 0.21 to 0.51] *p* = 0.31	*r* = −0.13 [CI: −0.46 to 0.18] *p* = 0.44	*r* = −0.22 [CI: −0.54 to 0.11] *p* = 0.19
SDNN (ms)	44 ± 14	*r* = 0.18 [CI: – 0.12 to 0.30] *p* = 0.29	*r* = 0.086 [CI: −0.28 to 0.43] *p* = 0.61	*r* = −0.11 [CI: – 0.48 to 0.26] *p* = 0.51
RMSSD (ms)	25 ± 13	0.46 [CI: 0.12 to 0.74] *p* = 0.004	*r* = −0.08 [CI: −0.43 to 0.26] *p* = 0.64	*r* = −0.50 [CI: −0.75 to 0.18] *p* = 0.0016
**Linear frequency-based HRV metrics**				
VLF (Ln)	6.6 ± 0.64	*r* = −0.03 [CI: −0.37 to 0.33] *p* = 0.85	*r* = 0.03 [CI: −0.35 to 0.41] *p* = 0.85	*r* = 0.1 [CI: −0.75 to 0.18] *p* = 0.0016
LF (Ln)	5.9 ± 0.60	*r* = 0.05 [CI: −0.27 to 0.35] *p* = 0.76	0.18 [CI: −0.17 to 0.50] *p* = 0.28	*r* = 0.02 [CI: −0.30 to 0.35] *p* = 0.90
HF (Ln)	5.2 ± 1.0	*r* = 0.36 [CI: 0.02 – 0.66] *p* = 0.028	*r* = −0.06 [CI: −0.41 to 0.29] *p* = 0.75	*r* = −0.42 [CI: −0.70 to −0.10] *p* = 0.009
LF/HF	0.69 ± 0.71	*r* = −0.42 [CI: – 0.68 to −0.08] *p* = 0.01	*r* = 0.19 [CI: −0.17 to 0.53] *p* = 0.27	*r* = 0.55 [CI: 0.23 to 0.78] *p* = 0.0004

The primary analysis concerns HRV with time to delivery (last column); correlations with GA at recording and GA at delivery are shown for completeness, *n* = 37.

GA, gestational age; HRV, heart rate variability; RRI, RR interval; SDNN, standard deviation of normal RRI; RMSSD, root mean square of successive differences between normal beats; HF, high frequency; LF, low frequency; VLF, very low frequency.

## Discussion

The purpose of this study was to study the possibility of finding an association between HRV with time to delivery. Our main finding was that there is a significant association between time to delivery and HRV. Specifically, we found significant correlations (*p* < 0.05) between RMSSD, HF, and LF/HF with time to delivery (Table 2).

Changes in HRV during pregnancy are well-documented (2, 6, 7, 18, 19). Most studies focused on investigating the changes in maternal HRV per trimester and they found that the sympathetic nervous system activity measured by using HRV increased in the third trimester from the second trimester (18–20). The increase in the sympathetic activity was assessed by the increase in LF and LF/HF (20). Contrary to the previous literature, our study focused only on HRV changes during the third trimester. The closest study to ours was done by Musa et al. (21), who compared HRV in labor with that in the third trimester (21). Musa et al. (21) found that LF, HF, and LF/HF increased during labor compared to the third trimester. The result related to HF is consistent with our finding, an increase in HF when the time of labor draws closer. However, our result related to LF/HF differs from Musa et al. (21), in which we found a decreasing LF/HF with closer labor.

Several methodological differences may explain this discrepancy. Musa et al. analyzed HRV in women already in active labor, whereas our study examined non-laboring women in the third trimester and correlated HRV with time to delivery. This difference in physiological state at the time of measurement may account for the contrasting autonomic patterns observed. In addition, Musa et al. relied solely on conventional ECG, while our analysis combined ECG and EHG-derived signals, which may introduce methodological variability. It is also worth noting that discrepancies in LF/HF findings were reported in previous studies (20) and such discrepancies are attributed to the complex time-sensitive processes that occur during pregnancy. Therefore, assessing HRV using a 5 min segment may yield results that differ from those obtained through longer-duration HRV recordings.

We speculate that the observed negative correlations between RMSSD and HF (Table 2) and time to delivery may reflect an increase in parasympathetic activity as labor approaches. This finding appears to contradict the widely reported norm of increased sympathetic activity near labor onset. However, we believe this result may reflect the inherent physiological complexity of labor. Rather than a linear progression of autonomic dominance, labor may be governed by a dynamic interplay between sympathetic and parasympathetic states, potentially involving a “sympathetic-parasympathetic alternation,” as the body prepares for the demands of delivery. Our findings may represent a transitional phase characterized by transient parasympathetic predominance prior to the sympathetic surge typically observed during active labor. We further speculate that the observed parasympathetic predominance could be modulated by neurohormonal factors. Oxytocin, which rises in late pregnancy and peaks during labor, has well-documented effects on the autonomic nervous system, including enhancing parasympathetic tone (22, 23). This neurophysiological link provides a plausible mechanism through which increasing oxytocin levels may shape maternal HRV patterns as labor approaches.

This study has several limitations. A substantial number of participants required induction or cesarean delivery, which necessitated their exclusion and reduced the final sample size by approximately half (from 84 to 37). The small sample size (*n* = 37) limited the statistical power. Furthermore, multiple HRV indices were tested without correction for multiple comparisons, increasing the risk of false correlations. The lack of data from the first and second trimesters limited our ability to explore whether early pregnancy HRV markers could serve as predictors of labor onset. Hence, these findings should be considered exploratory and validated in larger cohorts using appropriate statistical adjustments. The heterogeneity of the datasets is another limitation of this study, as maternal HRV was derived from both direct ECG and EHG signals. Although identical preprocessing and HRV extraction procedures were applied, the use of different modalities may have introduced bias and reduced comparability between groups.

The study can be extended by including fetal HRV which was found to be correlated with HRV (7, 24). Analysis of fetal HRV may provide further insights into the onset of labor and pregnancy outcomes. In addition, maternal HRV can be integrated with complementary physiological signals. For example, combining maternal and fetal HRV into a joint model may provide a more complete picture of maternal–fetal autonomic interactions and their role in the initiation of labor. For example, estimating maternal-fetal HR coupling patterns (25, 26) may provide further insights into the onset of labor. Similarly, multimodal monitoring approaches that incorporate maternal physiology (e.g., HRV, sleep, exercise) together with fetal HRV and wearable-derived signals could enhance predictive value beyond HRV alone. In addition, predictive modeling techniques such as regression analysis and machine learning could be applied to these multimodal datasets to identify complex, non-linear patterns that are not detectable with standard correlation analysis. Such approaches may ultimately enable the development of clinically useful prediction tools for the timing of labor onset.

## Conclusion

In this exploratory study, we found significant correlations between time to delivery and short-term HRV metrics. Specifically, HF and RMSSD were negatively correlated with the onset of labor, while LF/HF was significantly positively correlated. These associations may represent a transitional phase characterized by transient parasympathetic predominance prior to the sympathetic surge typically observed during active labor.

## Data Availability

The datasets presented in this article are not readily available because the Nordic dataset is publicly available online, however, Melbourne dataset is available on reasonable request. Requests to access the datasets should be directed to emersonkeenan@gmail.com.
